# Modern Campaign against Tuberculosis

**Published:** 1913-05-17

**Authors:** H. Hyslop Thomson

**Affiliations:** Tuberculosis Officer for the County of Herts; late Medical Superintendent of the Liverpool Sanatorium.


### MODERN CAMPAIGN AGAINST TUBERCULOSIS.

### The Work of a Tuberculosis Officer.

By H. HYSLOP THOMSON, M.D., D.P.H., Tuberculosis Officer for the County of Herts; late Medical Superintendent of the Liverpool Sanatorium.

The passing of the National Insurance Act of 1911 has created a new medical service, that of the tuberculosis officer. The duties of the tuberculosis officer have been clearly defined in the interim report of the Departmental Committee on Tuberculosis, but as the position and responsibilities of that official will vary considerably in different areas it is not possible in the present article to forecast correctly the lines along which future development may take place. It is desirable, however, to have some idea as to what such development should be. The tuberculosis service is at present in the making, and it will gradually become moulded into permanent form. If in the process of moulding a broad and comprehensive view be taken of the whole problem of tuberculosis, the future tuberculosis officer will constitute an important part of the public health service. Much, however, depends on the pioneer work in which the tuberculosis officer is at present engaged. His duties will prove to be many and varied, and they will call no less for tact and discrimination than for ability and clinical acumen. From the outset a high level of service with co-ordination of effort and the unification of methods of treatment should be aimed at.

### Administrative Duties.

At the outset the tuberculosis officer will be called upon to assist in organising a scheme for dealing with tuberculosis in his district, and a certain amount of administrative work will therefore fall to his share. The administrative duties will bring him into close touch with the medical officer of health of his district, and while it is desirable that the tuberculosis officer should have a certain amount of administrative independence and freedom of action, the medical officer of health being responsible for the administration of the public health work in the district, the scheme must be organised in consultation with him. In some districts the medical officer of health has been appointed tuberculosis officer, but this would appear to be quite unnecessary, as the designation medical officer of health is sufficiently wide and comprehensive to embrace the chief administrative duties relating to the problem of tuberculosis. Moreover, it will be a serious mistake if tuberculosis comes to be viewed as an administrative problem rather than as one requiring sound clinical knowledge. While experience in the administration of public health work will prove of value to the tuberculosis officer, the measure of his success will depend on his knowledge and experience of the disease and on the extent to which he realises that he must be a clinician first and all things else afterwards. Tuberculosis is a disease which in its frequent variation in type presents a many-sided clinical picture. It cannot, therefore, be bracketed with the acute specific diseases as one morbid entity and be dealt with by stereotyped methods of treatment. In his clinical work the tuberculosis officer should be independent, and his views on administration, based as they are on clinical knowledge of the disease, should receive due consideration. In this way the administrative experience of the medical officer of health and the clinical experience of the tuberculosis officer will provide an efficient combination for dealing with the problem of tuberculosis.

### Dispensary Duties.

The most important and responsible part of the work of the tuberculosis officer will be comprised in his clinical duties at the dispensary. Here he will be called upon to diagnose and recommend for suitable treatment the various clinical manifestations of tuberculosis as they are found in general practice. Suspected cases will be referred to him for a confirmatory diagnosis when the constitutional evidence and physical signs of infection with tubercle are slight, or indefinite. Young contacts will frequently have to be examined, and no more difficult problem exists in the whole field of medicine than the diagnosis of latent pulmonary tuberculosis in children.

An important part of the work at the dispensary will be the differentiation of type for sanatorium, hospital, dispensary, and domiciliary treatment, so that for each individual case the most suitable and beneficial method of treatment will be secured. This is one of the most important and responsible duties which the tuberculosis officer will be called upon to undertake, as it is one of the chief factors upon which depends the whole success of the modern campaign against tuberculosis. The result of the work at the dispensary in connection with diagnosis and differentiation of type will be that in the near future a gradual increase in the ratio of early to advanced cases will be observed.

The treatment of certain early and non-reactive types of the disease will also constitute part of the work of the tuberculosis officer at the dispensary. On taking up duties he will have to decide what form of tuberculin he will use and what method of administration he will adopt. In this he must not be unduly influenced by the advocacy of any particular school, but should rely on his own experience and reasoned judgment. His wisest course will probably be to use an exotoxic tuberculin until some degree of tolerance is produced, and then to follow immediately with a tuberculin containing both the endotoxin and the exotoxin of the human bacillus.

The work of the tuberculosis officer calls for a broad and liberal view with regard to the whole question of the treatment of tuberculosis. No effort should be made to advocate one method of treatment in opposition to another. The best results will be obtained if the various methods of treatment, including tuberculin, antiseptic inhalations, etc., are so co-ordinated at the dispensary that one will be employed to supplement the other, as occasion demands. In time the dispensary will come to be recognised as a central bureau for information and assistance with regard to all questions dealing with the problem of tuberculosis. In consequence, the views and expert opinion of the tuberculosis officer will frequently be consulted, and they will gradually become reflected, especially as to specific methods of treatment, in the views held by the general practitioners in his district.

### Relation to General Practitioner.

The work of the tuberculosis officer will frequently bring him into intimate professional relationship with the general practitioner, especially in rural districts. Much of the success of the new campaign against tuberculosis will depend upon the prompt recognition of the disease in latent form and in the early stage of onset, and to ensure this, friendly and intelligent co-operation between tuberculosis officer and general practitioner is absolutely essential. The advice of the tuberculosis officer will frequently be asked on matters relating to diagnosis and treatment. The general practitioner will draft patients to the dispensary, as he will quickly realise that when the home conditions preclude the possibility of efficient domiciliary treatment it will be through the medium of the dispensary that the treatment best suited to the requirements of his patient will be secured. On the other hand, it is to the general practitioner that the tuberculosis officer must look for his main supply of clinical material, and he will quickly come to realise that the more active the co-operation between them, the larger will be the number of cases referred to the dispensary. In many districts there will be a desire on the part of some of the medical practitioners to attend at the dispensary. The presence of the general practitioner at the dispensary during the hours of attendance to co-operate with the tuberculosis officer in the diagnosis and treatment of the disease will be mutually advantageous, especially in country districts. It will make for more efficient work, as those patients who are attending the dispensary for treatment will not be altogether removed from the supervision of the general practitioner, so that his interest in them will be retained. Indeed, it would be a mistake for the tuberculosis officer to endeavour to separate dispensary treatment from general medical practice and home treatment; they must be linked together if the best results are to be attained.

In many rural districts the general practitioner will look to the tuberculosis officer for guidance and instruction with regard to the administration of tuberculin. He will therefore become responsible, in part at least, for the views held by the general practitioners in his district and for the methods of treatment adopted. The tuberculosis officer thus holds a position of no small trust and responsibility. His aim should be to secure, as far as possible, the unification of methods of treatment in his district. It is only by this means that comparative results of any real value will be obtained and that our views with regard to the value of tuberculin in general practice will be advanced.

